# Randomised clinical trial of snus versus medicinal nicotine among smokers interested in product switching

**DOI:** 10.1136/tobaccocontrol-2014-052080

**Published:** 2015-05-19

**Authors:** Dorothy K Hatsukami, Herbert Severson, Amanda Anderson, Rachael Isaksson Vogel, Joni Jensen, Berry Broadbent, Sharon E Murphy, Steven Carmella, Stephen S Hecht

**Affiliations:** 1 Masonic Cancer Center, University of Minnesota, Minneapolis, Minnesota, USA; 2 Department of Psychiatry, University of Minnesota, Minneapolis, Minnesota, USA; 3 Oregon Research Institute, Eugene, Oregon, USA

**Keywords:** Harm Reduction, Non-cigarette tobacco products, Carcinogens

## Abstract

**Background:**

An essential component of evaluating potential modified risk tobacco products is to determine how consumers use the product and resulting effects on biomarkers of toxicant exposure.

**Study design:**

Cigarette smokers (n=391) recruited in Minnesota and Oregon were randomised to either snus or 4 mg nicotine gum for 12 weeks. Participants were instructed to completely switch from cigarettes to these products. Urine samples were collected to analyse for carcinogenic tobacco-specific nitrosamine metabolites (4-(methylnitrosamino)-1-(3-pyridyl)-1-butanol and N′-nitrosonornicotine and their glucuronides) and nicotine metabolites (total cotinine and nicotine equivalents) levels.

**Results:**

Of the 391 participants randomised, 52.9% were male, the mean±SD age was 43.9±12.5 years, baseline number of cigarettes/day was 18.0±6.5 and Fagerstrom Test for Nicotine Dependence score was 5.1±2.0. The mean±SD number of snus pouches used/week at week 6 prior to tapering was 39.1±24.0 and nicotine gum pieces used was 37.6±26.3. Dual use of cigarettes and these products were observed in 52.9% and 58.2% of those assigned to snus and nicotine gum, respectively, at week 12. The end of treatment biochemically verified (carbon monoxide, CO <6 ppm) 7-day avoidance of cigarettes was 21.9% in the snus group and 24.6% in the nicotine gum group. Toxicant exposure in the nicotine gum group was significantly less when compared to snus.

**Conclusions:**

Snus performed similarly to nicotine gum in cigarette smokers who were interested in completely switching to these products, but was associated with less satisfaction and greater toxicant exposure than nicotine gum.

**Trial registration number:**

NCT: 00710034.

## Introduction

In the USA and elsewhere, a low-nitrosamine smokeless tobacco known as snus is promoted as a complete or partial substitute for smoking. Differences in disease risk between cigarettes and snus have led some public health scientists to believe that if smokers completely switched to snus, then reduced tobacco-related mortality and morbidity would likely ensue.^1–5^ For example, the significant reduction in smoking among Swedish men leading to reductions in lung cancer, cardiovascular and all causes mortality, compared to other European Union countries,^2^ ^6^ has been attributed to substitution of cigarettes with snus. Smokers who switch to snus appear to have similar risks for cancer and cardiovascular disease as those who quit tobacco.^7^

Furthermore, several cross-sectional survey studies conducted in Scandinavia^2^ ^8^ ^9–11^ and the USA^12^ show that smokers who have ever used or used snus or snuff daily have a higher probability of quitting smoking than non-snus or non-snuff users. Survey studies conducted in Scandinavia^9^ ^10^ ^13^ and USA^14^ also show that snus or snuff, compared to medicinal nicotine, is more frequently used and/or leads to greater smoking cessation success, particularly among men.

Cross-sectional studies, however, do not distinguish whether or not the findings reflect differences in the characteristics of the population who use snus to quit smoking or the effects of the products themselves. A randomised clinical trial would help determine if snus compared to medicinal nicotine leads to higher rates of stopping smoking as a result of complete switching or a greater reduction in smoking and consequent reduction in exposure to harmful constituents. Besides a small pilot study that we conducted,^15^ no such clinical trial has been reported in the literature.

The primary goal of this study was to compare snus versus nicotine gum on the extent to which smokers can completely switch to these products, the pattern of product use and effects on biomarkers of exposure. The secondary goals were to compare the effects of both products on withdrawal symptom relief, product evaluation and adverse events.

## Methods

### Participant recruitment

Cigarette smokers interested in completely switching to snus or nicotine gum were recruited from Minneapolis/St Paul, Minnesota, and Eugene, Oregon, between May 2010 and May 2013, using internet and local media advertisements. Participants were followed through June 2014. Interested smokers who telephoned the research clinics were briefly informed about the study and screened for eligibility. Eligibility criteria included: (1) 18–70 years old; (2) smoking at least 10 cigarettes daily for the past year, (3) in good physical and mental health (no unstable or untreated medical or psychiatric condition); (4) no contraindications for medicinal nicotine; (5) no regular use of other nicotine or tobacco products; and (6) if female, not pregnant or nursing. Eligible participants attended the research clinic for an orientation visit, provided informed consent and engaged in more thorough screening, including assessment of their medical and tobacco use history, and nicotine dependence, using the Fagerstrom Test for Nicotine Dependence,^16^ and pregnancy testing. This study was approved by each site's Institutional Review Board, and a Data and Safety Monitoring Board (DSMB) met annually to monitor study progress and adverse events.

### Products

The oral tobacco product chosen was Camel snus (Winterchill and Robust, 2.5 and 2.6 mg nicotine/pouch, respectively, distributed by Reynolds American Inc). These snus products were chosen because of the higher levels of unprotonated nicotine in them compared to other US manufactured snus products and prior research showing that suppression of smoking is greater with oral non-combusted products with higher nicotine levels.^17^ Participants who experienced adverse effects from these doses were provided Frost or Mellow (1.5 and 1.3 mg nicotine/pouch, respectively). A Swedish snus product with even higher levels of nicotine may have been preferable to compare with the Swedish experience. However, our prior preference study showed that no smoker chose the Swedish snus (General Snus).^17^ Nicotine gum (4 mg Nicorette distributed by GlaxoSmithKline) was chosen as the medicinal nicotine product, and participants who experienced adverse effects were down-titrated to 2 mg nicotine gum.

### Study design

Participants were informed that the study examined the effects of snus versus nicotine gum on smoking behaviour and potential health effects. During an initial 1-week baseline data collection, participants reported the number of cigarettes and symptoms associated with withdrawal (Minnesota Nicotine Withdrawal Scale, MNWS^18^ ^19^) using an Interactive Voice Response (IVR) system at the end of each day. Other baseline measures were collected at the clinic visit (see below).

After the baseline period, participants were randomised to one of the two treatment conditions for 12 weeks. Randomisation lists (separate for each site) used block sizes of 10. In both conditions, participants were encouraged to use only the assigned product and at least 6–8 pieces a day for about 30 min each or optimally every 1–2 h, and more if necessary. Per instructions described for nicotine gum, all participants (regardless of randomisation group) were advised to reduce consumption by half during weeks 7–9 and three-quarters between weeks 10–12 to minimise the risk of persistent use or withdrawal from the products when they were no longer provided. Although any non-assigned tobacco or medicinal product use was discouraged, accurate self-report was stressed.

After product assignment, participants returned to the clinic weekly for 2 weeks and bi-weekly for the remaining 10 weeks. At the clinic visits, participants were asked to return all unused product (with the amount used and returned logged in our records) and new products were dispensed, outcome measures collected and brief 10 min standardised behavioural and compliance counselling was provided. The topics covered in the session were based on the *Clearing the Air* treatment manual^20^ and provided to each participant. Trained counsellors were given a worksheet that described the topics to be covered for each session. Total number of clinic visits was 8 with one phone call at week 10. Follow-up sessions occurred at 13 and 26 weeks from the onset of treatment, with two follow-up calls at weeks 19 and 39 (not included in the analysis). Participants were reimbursed a total of $360 for visit attendance and blood draws.

### Measures

Throughout treatment, participants reported product use and any cigarettes smoked on a daily basis using the IVR system. Other measures collected at the baseline and treatment clinic visits included the past week's MNWS, adverse events, vitals and alveolar carbon monoxide (CO). Responses to products were measured using Product Evaluation Scale (Weeks 1, 4 and 12),^21^ a 7-point Likert-type scale modified from the Cigarette Evaluation Scale.^22^ Scale scores addressed four factors: reflecting product satisfaction, psychological reward, sensation in mouth and aversion.^23^

Biomarkers were collected at baseline and week 4 (to maximise the number of data points, prior to relapse or drop-outs). Biomarkers included measures of nicotine exposure: total cotinine^24^ and total nicotine equivalents (the sum of total nicotine, total cotinine and total 3′hydroxycotinine, TNE)^25^ In addition, biomarkers included measures of tobacco-specific carcinogens: (1) urinary 4-(methylnitrosamino)-1-(3-pyridyl)-1-butanol and its glucuronides (total NNAL),^26^ which are metabolites of 4-(methylnitrosamino)-1-(3-pyridyl)-1-butanone (NNK) and (2) *N′*-nitrosonornicotine (NNN) and its glucuronides (total NNN),^27^ which reflect the uptake of NNN.

At follow-up, 26 weeks after start of treatment, smoking abstinence and use of any other tobacco or medicinal nicotine products were assessed using time line follow-back,^28^ and biochemical verification was obtained.

### Sample size calculation

The planned sample size for this study was 400 participants, powered to detect differences between treatment groups in the point prevalence (7-day cigarette avoidance) at the completion of treatment (week 12). Group sample sizes of 200 in each group would achieve at least 80% power to detect an absolute difference between 7-day cigarette avoidance rates of 10%, if avoidance rates are 12% and 22%, using nicotine gum and snus,^9^ respectively, using a two-sided Z test and significance level of 0.05.

### Analytic approach

Participants’ baseline characteristics were compared by product group (nicotine gum vs snus). Discrete variables were analysed using Pearson's χ^2^ test or Fisher's exact test. Continuous variables were analysed using t tests and Wilcoxon Rank Sum tests as appropriate.

We conducted an intention-to-treat analysis. Biomarkers of exposure were analysed on the natural log scale to ensure normality and comparisons between baseline and week 4 were conducted using repeated measures linear regression models. All other continuous outcomes with repeated measures from baseline through treatment were analysed using linear mixed models with fixed effects for site, product, week, interaction between product and week, and a random effect for participant. Least squares means and 95% CIs are presented unless otherwise noted. The differences in the 7-day point prevalence and continuous cigarette avoidance at weeks 12 and 26, or use of any nicotine-containing product at week 26, between product groups, were evaluated using Pearson's χ^2^ tests. Study drop-outs were assumed to have started smoking unless otherwise noted. Smoking avoidance was verified using CO levels (<6 ppm) and avoidance of all nicotine-containing products was verified using cotinine (<35 ng/mL). A sensitivity analysis was performed to confirm that the results were similar when including only participants with follow-up data (as compared to the intention-to-treat analysis), and the conclusions were the same.

Data were analysed using SAS V.9.3 (Cary, North Carolina, USA), and p values <0.05 were considered statistically significant.

## Results

### Participant characteristics

**Figure 1:**
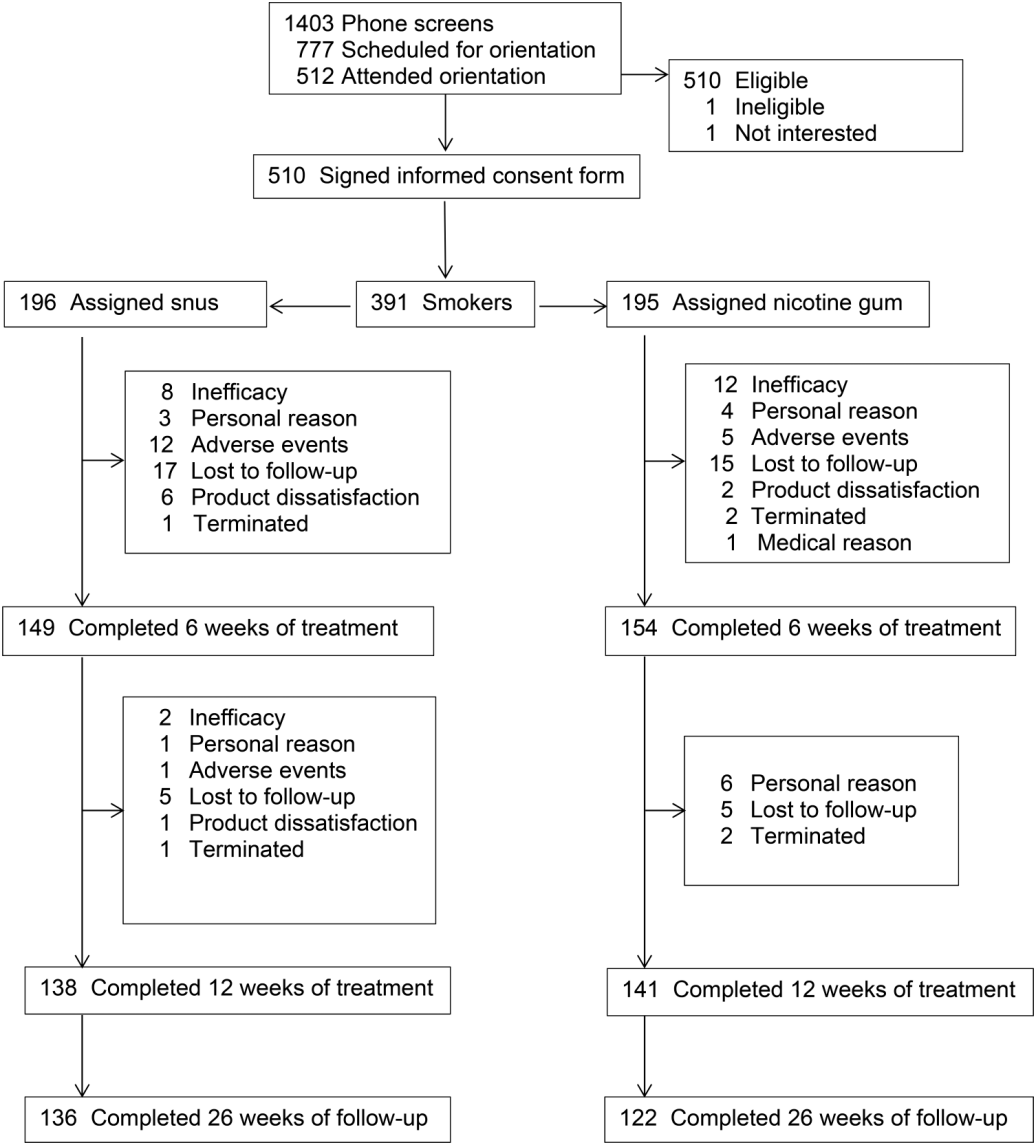
Flow of participants in study.

With approval from the DSMB, recruitment was stopped early due to time constraints; a total of 391 participants were enrolled in the study. Figure 1 shows the disposition of the participants in the study and table 1 provides baseline demographic and tobacco use history information. No significant differences were observed by product group. However, site differences were observed with participants in Minnesota being older (46.5±11.7 vs 41.5±12.8, p<0.0001) and smoking more cigarettes per day (19.3±7.3 vs 16.9±5.5, p=0.0003).

**Table 1:** Baseline demographics and smoking history (percent or mean±SD) of participants (N=391) by treatment group

	Overall		Nicotine gum		Camel snus	
Age (years)	391	43.9±12.5	195	44.7±12.5	196	43.2±12.5
Female	184	47.1%	95	48.7%	89	45.4%
Non-Hispanic whites	320	81.8%	165	84.6%	155	79.1%
Education						
Some high school	25	6.4%	11	5.6%	14	7.1%
High school graduate or equivalent	107	27.4%	52	26.7%	55	28.1%
Some college/2-year degree	192	49.1%	99	50.8%	93	47.5%
College graduate/4-year degree	51	13.0%	25	12.8%	26	13.3%
Graduate	16	4.1%	8	4.1%	8	4.1%
Cigarettes per day	391	18.0±6.5	195	18.3±6.8	196	17.8±6.2
Age smoking first cigarette (years)	386	14.3±4.2	194	14.4±4.3	192	14.2±4.1
Age becoming a regular smoker (years)	386	17.6±4.8	194	17.8±5.3	192	17.3±4.1
Ever tried smokeless tobacco	171	44.4%	79	40.7%	92	48.2%
Ever tried nicotine gum	117	29.9%	60	30.8%	57	29.1%
Number of quit attempts						
0–2	140	36.3%	68	35.1%	72	37.5%
3–5	141	36.5%	74	38.1%	67	34.9%
6–10	57	14.8%	26	13.4%	31	16.2%
11–20	48	12.4%	26	13.4%	22	11.5%
FTND	390	5.1±2.0	195	5.0±1.9	195	5.2±2.0

FTND, Fagerstrom Test for Nicotine Dependence.

Where data is missing, N is less than 391.

### Product use

**Figure 2:**
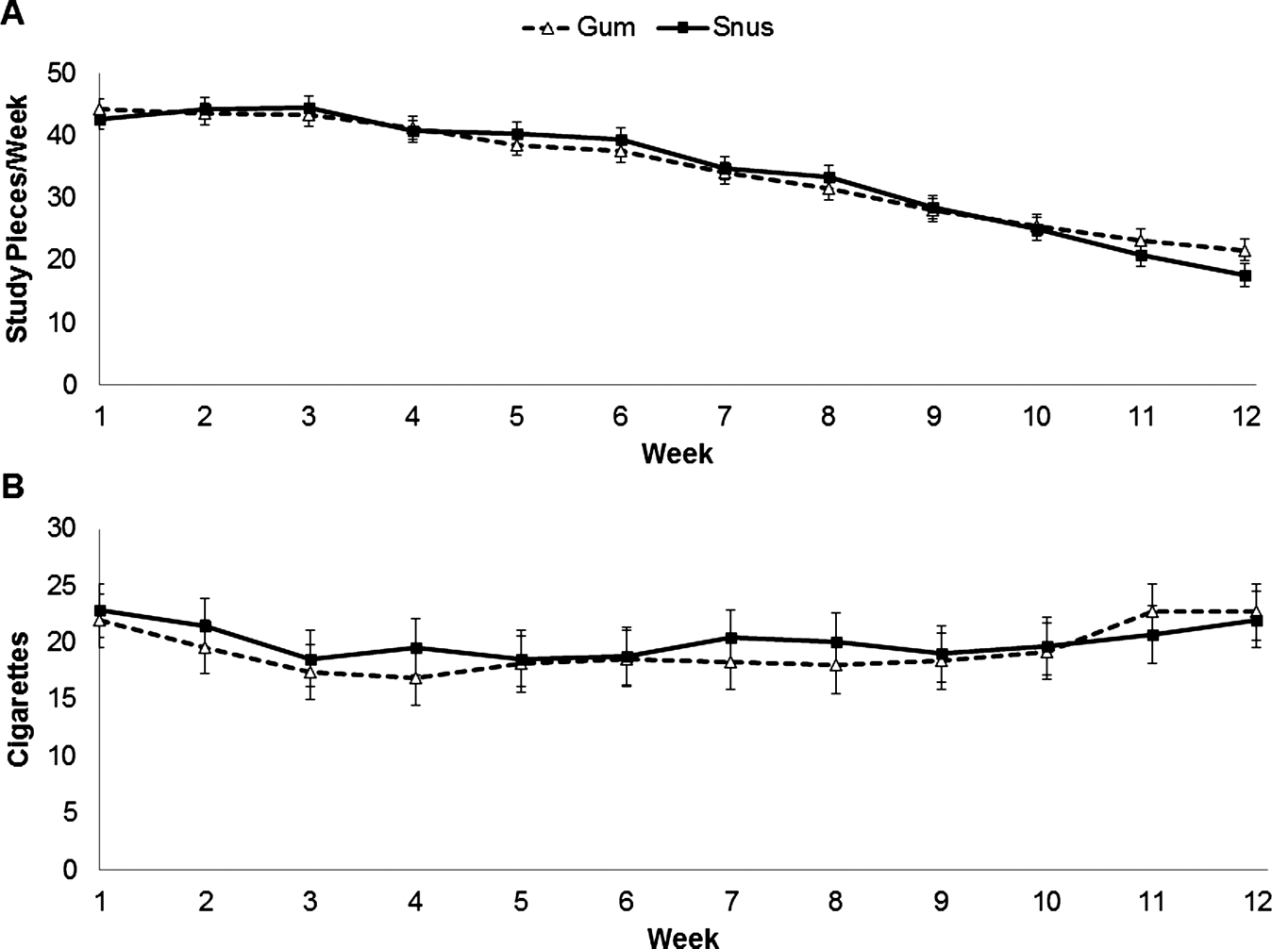
Mean (SD) amount of product use (A) and amount of cigarette use among dual users by product group (B).

Based on the IVR, the proportion of participants using the study products did not statistically significantly differ between assigned products at any week during the treatment period; use ranged from 99% for snus and 100% for gum at week 1 and gradually declined to 80% for snus and 87% for gum at week 12. The lowest proportion reporting using each study product was at week 11 for gum (82%) and week 12 for snus (80%). Figure 2A shows the mean product use per week. Although significant week effects were observed (p<0.0001), no other significant differences were observed.

Table 2 shows the proportion of participants using only the study product, smoking cigarettes plus study product, smoking cigarettes only and using neither study product nor cigarettes at week 6 (prior to taper) and week 12 (end of treatment). No significant differences were observed across study products. Use of other products during the treatment was minimal; less than 5% of participants reported use of cigars, e-cigarettes, smokeless tobacco or other nicotine replacement therapies.

**Table 2:** Proportion of participants using different products during study product assignment†

	Week 6**				Week 12***			
	Gum (N=153)		Snus (N=149)		Gum (N=141)		Snus (N=138)	
	N	Per cent	N	Per cent	N	Per cent	N	Per cent
Product only	56	36.6	56	37.6	40	28.4	37	26.8
Cigarette+product use	79	51.6	82	55.0	82	58.2	73	52.9
Cigarette only	9	5.9	5	3.4	6	4.3	16	11.6
Neither cigarette nor product use	9	5.9	6	4.0	13	9.2	12	8.7

**p=0.627, ***p=0.158.

†Based on Interactive Voice Response System.

Focusing on dual use, the proportion of dual users varied during treatment for snus (53–77%) and nicotine gum (48–79%), with the highest percentage occurring earlier in the experimental period. Figure 2B shows the mean number of usual brand cigarettes used *per week*. Significant effects were only observed for site (p<0.0001; greater in Oregon than Minnesota) and study week (p=0.019). When correlating the extent of product use with cigarettes smoked, during week 1, more cigarette use was weakly associated with lower nicotine gum and snus use (r=−0.17, p=0.018 and r=−0.27, p=0.0003); this continued for those in the snus group through week 5 (r=−0.21 to −0.24, p values=0.001–0.006). In the gum group, higher cigarette use in the latter weeks (9–12) was weakly associated with higher product use (r=0.18–0.31, p values=0.002–0.029).

### Biomarkers of exposure

For cotinine, significant reductions were observed from baseline to week 4 for nicotine gum and snus (both p<0.0001). No significant effects by study product or site were observed at week 4 after adjustment for baseline values. TNE results were similar. For urinary total NNAL, significant reductions were observed from baseline to week 4 for nicotine gum (p<0.0001), but not snus. Significant differences at week 4 by study product (p<0.0001) were observed; nicotine gum users showing greater change than snus users.

Table 3 shows total NNAL, total cotinine and TNE values for assigned product only or dual users (cigarettes plus assigned product) among those with baseline as well as week 4 values. Comparisons of week 4 values were adjusted for baseline biomarker values and site, and corrected for multiple comparisons. Among product only and dual users, total NNAL was significantly higher among snus versus nicotine gum users (p<0.001 and 0.005, respectively). While no significant differences were observed for the snus only and snus dual users, significantly lower levels of total NNAL were observed in the nicotine gum only versus gum dual users (p=0.001). Although no other comparisons were statistically significant for total cotinine, significantly lower TNE levels for snus only versus dual users (p=0.042) were observed.

**Table 3:** Urinary biomarkers by treatment and study product only or dual use of study product and cigarettes at week 4

	Nicotine gum				Snus			
	Baseline		Week 4		Baseline		Week 4	
	N*	Mean (SD)	N*	Mean (SD)	N*	Mean (SD)	N*	Mean (SD)
Total NNAL (pmol/mg creatinine)								
Study product only†	58	1.39 (1.04)	58	0.30 (0.39)	52	1.28 (0.94)	52	1.34 (1.42)
Dual users	96	1.58 (1.19)	96	1.11 (1.00)	96	1.47 (1.55)	96	1.55 (1.67)
Total cotinine (ng/mL)								
Study product only†	59	3481 (1839)	59	2052 (2342)	53	3385 (1672)	53	2152 (2005)
Dual users	97	3572 (2297)	97	2838 (2229)	100	3359 (2145)	100	3079 (2398)
Total nicotine equivalent† (nmol/mL)								
Study product only†	59	63.6 (40.9)	59	36.0 (42.1)	53	59.5 (32.3)	53	35.6 (31.0)
Dual users	97	65.8 (42.1)	97	51.2 (41.9)	100	66.2 (49.3)	100	55.7 (43.0)
Total NNN* (pmol/mg creatinine)								
Study product only†	24	0.06 (0.07)	24	0.01 (0.01)	18	0.06 (0.06)	18	0.06 (0.07)
Dual users	25	0.09 (0.09)	25	0.05 (0.05)	23	0.13 (0.22)	23	0.11 (0.10)

*Lower number of participants is due to analysis of a subset of participants.

†Carbon monoxide (CO) verified (<6 ppm).

Owing to costs, for total NNN, a random selection of participants who reported using >30 pieces/week of the assigned product during weeks 2–4 were selected, and dual users reported reducing their smoking 35–85% at week 4 compared to baseline (table 3). Significantly lower urinary total NNN were observed among those who only used nicotine gum compared to snus (p<0.0001), but no significant differences were observed across dual users. Nicotine gum only users had significantly lower total NNN compared to gum dual users (p=0.008), but no differences were observed between snus only compared to snus dual users.

### Effect on subjective responses: withdrawal and product evaluation

For withdrawal symptoms (craving excluded), although significant study week effects were observed (p<0.0001), with a significant increase occurring from Baseline to week 1 (p<0.0001), no significant product, or product by study week effects, were evident. There was a statistically significant difference by site (p=0.021), with those participants in Minnesota experiencing lower withdrawal. In addition, craving significantly decreased over time (p<0.0001), though it did not differ significantly by product or site.

For the product Satisfaction and Psychological Reward scales, significant study week (p<0.0001 for both scales), product (p<0.0001 and p=0.009, respectively) and product by study week effects (p=0.001 and p=0.005, respectively), were observed. Usual brand cigarettes were more satisfying and psychologically rewarding than either of the products (p<0.0001 both products and scales, respectively), and those assigned to nicotine gum reported greater satisfaction and psychological reward than snus users. Site differences were also observed (p≤0.0001), with those users in Minnesota reporting higher scale scores. Likewise, for Sensation in Mouth, study week (p<0.0001), product (p<0.0001) and product by study week (p<0.0001), effects were statistically significant, though site was not. Gum users reported higher scores on this scale than snus users, and usual brand cigarettes were rated lower than either product (p<0.0001 and p=0.007, respectively), indicating that mouth sensation may not be as highly associated with cigarette smoking. Although no product effects were found for Aversion, study week effects (p<0.0001) showed significantly higher scores for the study products compared to usual brand cigarettes and a significant site effect (p=0.003), with those in Minnesota reporting greater aversion to the products.

The adverse events that were experienced were typical of those found with nicotine gum, and only a few differences were observed between the two study products (see online supplemental table). It appears that gum and snus led to experiences of dry mouth, excessive salivation, dizziness, light-headedness (snus users), nausea, stomach aches, sore jaw, belching, hiccups, sore throat, mouth sores and, interestingly, a reduction in anxiety, and for snus users, a reduction in reported headaches. Significant differences were observed between snus and nicotine gum for excessive salivation (p<0.0001), headaches (p=0.022) and mouth sores (p=0.020); more snus users experienced excessive salivation and mouth sores but fewer experienced headaches compared to nicotine gum users.

### Cigarette and product use at the end of treatment and follow-up

In the intent-to-treat analysis, at the end of the treatment phase (week 12), no significant differences were observed for 7-day cigarette avoidance between nicotine gum and snus (24.6% vs 21.9%, respectively) or for continuous cigarette avoidance from week 2 to end of treatment (9.7% vs 5.6%, respectively). Similarly, at week 26, no significant differences between nicotine gum and snus were observed for 7-day cigarette avoidance (15.4% vs 11.2%, respectively) and continuous cigarette avoidance (5.1% vs 2.6%, respectively), or for point prevalence (9.7% vs 5.6%, respectively) and continuous (3.6% vs 2.0%, respectively) avoidance of all nicotine products. There were no statistically significant differences by site for any of these outcomes.

With regard to continued product use, among those assigned to nicotine gum and participated at the 26 week follow-up, approximately 6.0% self-reported gum use only and 6.8% reported gum and cigarettes use. The rates were 14.9% and 11.6%, respectively, for snus use among those assigned to snus. The distribution of these rates was significantly different between snus and gum (p=0.006), with higher rates of continued snus use among those assigned to this product.

## Discussion

The results showed no significant differences between those assigned to medicinal nicotine vs snus in amount of product use, levels of cotinine attained, the extent to which the product substituted for smoking and rates of avoidance of cigarettes or any nicotine containing products. Furthermore, there were no differences in suppression of withdrawal from cigarettes. However, the nicotine gum users reported more satisfaction and psychological reward from the product, experienced less carcinogen exposure during the time of peak use, and had fewer participants who continued product use compared to the snus users. These findings suggest that snus performs no better than nicotine gum as a cigarette substitute, has less appeal, is more toxic and is associated with higher rates of prolonged use.

The results from the current study replicate our prior pilot study, which showed that snus, at doses similar to nicotine gum, is no better than medicinal nicotine in the extent to which complete substitution occurred.^15^ Similar to the present study, Kotlyar *et al*^15^ showed higher levels of urinary total NNAL when smokers substituted snus compared to medicinal nicotine for cigarettes. However, while the prior study showed a significant reduction in total NNAL when switching to snus, in the current study, no reduction was observed. This difference across studies may be attributed to the roughly threefold increase in NNK/pouch between 2010 and 2012–2013 (from 0.15 to 0.49 µg/pouch). With regard to NNN, in the Kotlyar *et al*,^15^ study no significant reduction was found when smokers switched to snus, which was similar to our findings from the present study. Furthermore, significant reductions in total NNN were observed with nicotine gum use. However, unlike the Kotlyar study, which observed no group differences, the present study observed greater reductions in total NNN in nicotine gum users, potentially due to the analysis of a subset of the participants in the current study or a larger sample size.

Contrary to the results from survey studies in Scandinavian countries, in this study, snus was associated with less positive subjective responses compared to medicinal nicotine. This finding is concordant with other North American clinical and survey studies showing medicinal nicotine products being preferred over oral tobacco products such as snus or dissolvable tobacco products.^29–31^ On the other hand, in a brief clinical study conducted in New Zealand, heavy smokers reported greater preference for Zonnic (an oral nicotine sachet of 4 mg of nicotine bound to beads) and Swedish snus, over nicotine gum, for use in quitting or reducing cigarettes.^32^

Unlike survey studies in Scandinavian countries, which have demonstrated high interest in snus among men, US surveys show relatively less interest in snus. In one survey, although 29.9% of male smokers reported ever trying snus, only 4.2% reported current use. Current use was negligible in former smokers. The authors concluded that the low rate of adoption of snus in the US market signifies that this product is unlikely to be a significant harm reduction agent or to lead to public health harm.^33^ Other survey studies have also demonstrated low interest in the use of snus in the USA.^34^ ^35^ Perhaps the lack of US interest in snus as a substitute for cigarettes is why recruitment for this study took longer than anticipated.

The results also show that a substantial number of smokers motivated to switch completely to an alternative product become dual users during the treatment period. This finding could be related to the low levels of free nicotine in our chosen product, the slower rate of absorption relative to cigarettes and the lack of the sensory aspects of smoking, attributes which lead to being a poor substitute for cigarettes. Human laboratory and short-term product cross-over studies have demonstrated that although medicinal nicotine and snus sold in the US suppressed cigarette abstinence-induced craving, usual brand cigarettes led to substantially greater suppression of craving. Furthermore, acceptability of these products was substantially lower than usual brand cigarettes and, in one study, was more in line with the sham smoking condition, leading the authors to conclude that these products are unlikely to completely substitute for cigarettes.^36^ ^37^ In one study, even when smokers were offered a Swedish snus product with a high nicotine dose, no one chose this product for use during smoking cessation after sampling it.^17^

The trend toward more favourable responses to nicotine gum points to two observations. First, the use of 4 mg medicinal nicotine as a substitute for cigarette smoking and a harm reduction agent should be considered. More recently, an application for Swedish snus as a modified risk tobacco product has been submitted (Docket ID: FDA-2014-N-1051), yet medicinal nicotine gum more clearly results in reduced exposures to toxicants. Second, other minor tobacco alkaloids have been considered as potentially contributing to the reinforcing effects of tobacco products,^38^ ^39^ the results from this study suggest that these constituents may or may not play an important role in tobacco self-administration or response. That is, the amount of self-administration was similar across products (perhaps due to instructions for amount of use), but sustained use was higher with snus. Rat studies tend to show smokeless tobacco extracts do not enhance nicotine effects compared to similar doses of nicotine alone.^40^

There are several limitations to this study: (1) potential lack of generalisability to a general population of smokers because we examined smokers interested in trying an alternative product in a clinic setting, (2) testing only one snus product, which has lower levels of nicotine and higher TSNA than some of the Swedish snus products, (3) encouragement to use a specified number of pieces of each of the products; (4) implementation of a tapering period, which might have constrained substitution behaviour; and (5) not examining the data by gender (eg, men as opposed to women may respond more positively to snus).

Nonetheless, these results suggest that, in the USA, snus may not succeed in substituting for cigarettes because of lack of interest and appeal, even when compared to medicinal nicotine. The differences in responses to snus-like products across different countries and even different sites emphasise the need to take into account the cultural context in which a product is being marketed and used. Because medicinal nicotine performed no differently or even better on some outcome variables than the snus that we tested, and had substantially less carcinogen exposure, even when this snus product alone is compared to dual nicotine gum and cigarette users, it is prudent to recommend medicinal nicotine over snus, particularly in the USA, for those who want to reduce or completely switch to another product.

**What this paper adds**

**What is already known on this topic**

- Switching from cigarettes to snus products has been described as a harm reduction method for smokers who are unable or unwilling to quit smoking.
- In Sweden, switching from cigarettes to snus has resulted in a significant reduction in tobacco-related health risks.

**What important gaps in knowledge exist on this topic**

- So far, no clinical trial has examined the effects of snus versus medicinal nicotine on pattern of product use, exposure biomarkers, subjective responses and the extent of complete switching.
- Furthermore, the generalisability of results from Sweden to other countries is unknown.

**What this study adds**

- In this US-based study, US-marketed snus performed no better than nicotine gum in cigarette smokers who were interested in completely switching to these products, but was associated with greater toxicant exposure and less satisfaction than nicotine gum.
- Generalising harm reduction effects observed in Sweden to another country may be limited.

## Supplementary Material

Web supplement
