# Supplementary material for: Randomised clinical trial of snus versus medicinal nicotine among smokers interested in product switching
Source: Tob Control. 2015 May 19;25(3):267–74. doi: 10.1136/tobaccocontrol-2014-052080 (PMC4785094; doi:10.1136/tobaccocontrol-2014-052080)
Supplement: Web supplement [file tobaccocontrol-2014-052080-s1.pdf]

Supplemental Table. Adverse events (AEs) at baseline and during the study.*

	Baseline**				During Study***				
	Gum (N=195)		*Snus (N=196)*		*Gum (N=195)*		*Snus (N=196)*		
**AE**	**Mild**	**Mod-Severe**	**Mild**	**Mod-Severe**	**Mild**	**Mod-Severe**	**Mild**	**Mod-Severe**	**p-value**
Dry Mouth	29	4	35	4	40	2	48	7	0.138
Excessive Salivation	2	2	5	4	37	4	57	19	<0.0001
Headache	25	16	25	19	26	6	13	14	0.022
Dizziness	11	1	10	2	21	1	23	1	0.936
Lightheaded	11	0	11	1	14	1	20	1	0.684
Nausea	1	2	2	2	35	8	48	13	0.121
Belching	8	1	9	0	34	3	22	2	0.166
Hiccups	13	0	7	0	45	4	38	4	0.645
Sore Throat	13	2	15	0	26	6	34	2	0.211
Mouth Sores	1	0	3	0	31	1	37	9	0.020
Sleep Disturbance	28	21	31	13	23	12	22	6	0.330
Anxiety	43	8	59	8	16	7	18	3	0.442

*AE was listed if it was reported by >5% of the sample. Because of so few severe AEs, they were combined with moderate severity.
All reported AE's at baseline, regardless of cause.*Maximum severity across the study (each person counted once), among those AEs noted as definitely related, possibly related or unknown cause. Those AEs with cause noted as unrelated were not included.
